# Exploring the Unmet Need for Preconception Care and Its Effect on Biomarkers Among Married Nulliparous Women in Guntur, Andhra Pradesh: A Community-Based Cross-Sectional Study

**DOI:** 10.7759/cureus.100257

**Published:** 2025-12-28

**Authors:** Mudireddy Nandini, Venkatashiva Reddy B, Ramireddy Gari Likhitha, Vijay Kishore A, Rajeev Aravindakshan, Ishant Kumar, Harshita Choudhary

**Affiliations:** 1 Department of Community and Family Medicine, All India Institute of Medical Sciences Mangalagiri, Mangalagiri, IND

**Keywords:** hypothyroidism, married, nulliparous, preconception care, serum biomarkers

## Abstract

Background: In India, preconception care (PCC) is vital because the high rate of unplanned pregnancies raises the risk of complications for both mothers and newborns, and many young women lack adequate vaccination coverage before pregnancy. PCC is crucial for reducing adverse maternal-fetal outcomes. Still, knowledge and utilization of PCC remain very limited in many low- and middle-income settings. Evidence on the unmet need of PCC along with vaccination, lifestyle-related PCC, and biomarker associations in young women is limited in the Indian setting. This study explores the prevalence of PCC along with vaccination and nutrition-related PCC in nulliparous women.

Methodology: A community-based cross-sectional study was conducted from February to April 2025 in the outreach area of a primary health center in Guntur, Andhra Pradesh. Married nulliparous women aged 18-30 years were recruited using systematic sampling (n = 220). Data collection tools used included a pretested semi-structured questionnaire (including FIGO preconception checklist) and anthropometry. Statistical analysis included χ² tests and multivariable logistic regression to identify predictors of PCC awareness (IBM SPSS Statistics for Windows, Version 29 (Released 2024; IBM Corp., Armonk, New York, United States); p < 0.05 as statistically significant).

Results*:* Among 220 participants, only 49 (22.3%) had heard of PCC, and 14 (6.4%) were aware of vaccination-related PCC. Multivariable analysis showed that occupation of working vs. housewife with AOR 0.349 and 95% CI (0.062-1.968) and access to multiple information sources with AOR 0.007 and 95%CI (0.001-0.0035) were significant predictors of PCC awareness.

Conclusions: Awareness and utilization of vaccination and lifestyle-related PCC remain significantly low among young nulliparous women in South India. Integrating vaccination and lifestyle counseling into routine PCC at primary health centers, supported by frontline worker training, policy mandates, and community awareness campaigns targeting young women, could bridge critical gaps and improve pre-pregnancy health outcomes.

## Introduction

According to the World Health Organization (WHO), preconception care (PCC) is defined as the provision of biomedical, behavioral and social health interventions to women and couples before conception occurs [1]. PCC awareness was defined as having heard of PCC prior to the survey and unmet need for PCC was defined as lack of access to or use of health services addressing vaccination, lifestyle, or medical conditions as outlined in the FIGO checklist [2]. PCC represents a critical yet often overlooked component of maternal health. It includes delivery of preventive, promotive, and curative interventions before conception, to optimize maternal health and improve pregnancy outcomes, reducing neonatal morbidity and mortality. The WHO has emphasized PCC since 2013 [3]. PCC remains inadequately integrated into health systems across many low- and middle-income countries, including India.

India has made significant progress in reducing maternal and neonatal mortality, with the maternal mortality ratio declining to 97 per 100,000 live births (SRS 2018-20) [4] and neonatal mortality dropping to 23 per 1,000 live births (SRS 2019) [5]. However, these improvements have plateaued in recent years, and preventable causes continue to contribute to poor outcomes. Unplanned pregnancies, untreated chronic illnesses, inadequate vaccination coverage, and lifestyle-related risk factors persist as barriers to achieving optimal maternal and newborn health. The lack of planning for pregnancy and low rates of contraception use are of concern, as women are underprepared to enter pregnancy [6]. Addressing these determinants through PCC may accelerate progress toward national and global health targets.

While several studies have examined the role of nutrition within PCC [7-10], other domains, such as vaccination, lifestyle modification, and screening for chronic conditions, remain less explored. Enhancing preconception health has been shown to lead to better outcomes for both the fetus and the mother. Promoting health before conception had multiple benefits, such as increased utilization of antenatal care and reduced neonatal mortality rates. Preconception health was understood as a comprehensive concept that included managing chronic diseases, ensuring optimal nutrition, adequate folic acid intake, maintaining a healthy body weight, adopting healthy lifestyles, and receiving appropriate vaccinations. At the time, PCC activities in India mostly focused on family planning and peri-conceptual folic acid supplementation, with limited coverage and inadequate tracking within routine health management information systems. Although there had been recall-based studies on PCC received by pregnant women [11,12], there remained a gap in knowledge regarding the utilization of PCC by nulligravida women in India. This study explores the prevalence of unmet need for PCC along with vaccination and nutrition-related PCC among nulliparous women. This study estimates the lack of knowledge on existing medical care and the relationship of HbA1C and thyroid-stimulating hormone (TSH) with the unmet need of PCC.

## Materials and methods

PCC awareness is defined as knowing PCC services and their importance. PCC utilization refers to attending at least one PCC consultation within the past 12 months. Unmet need is defined as the expressed desire for PCC that has not been received during the same period. Stress levels were measured using a scale from 1 to 9, with higher scores indicating greater perceived stress.

This is a community-based cross-sectional study and was carried out in the outreach area of a primary health center (PHC) located in Guntur district, Andhra Pradesh, South India. The study protocol was approved by the Institutional Ethics Committee (AIIMS/MG/IEC/2024-25/52 Dated:19-09-2024). The study was conducted over two months from February to April 2025. The sample size was calculated using the formula (n = 4pq / d^2) assuming a prevalence of PCC utilization at 14.5% [10] from prior Indian studies, with 5% absolute precision, 95% confidence level, and 10% non-response rate. The final sample size was 220 participants. Systematic sampling was employed to select eligible women.

The study population consisted of married nulligravida women aged 18-30 years who resided in the PHC catchment area for at least three months. Nulligravida women who were currently married and living with their husbands and were residents of the study area for at least three months were included in the study. Women with intellectual disabilities or serious illness at the time of survey, and women with known reproductive tract pathology, HIV, or malignancy, and non-consenting individuals were excluded from the study. All the eligible mothers are informed prior about the study using a patient information sheet. Informed written consent was obtained from the study participants who were willing to participate. Confidentiality and anonymity were strictly maintained throughout data collection and analysis.

Data collection was done using a pretested semi-structured questionnaire adapted from the International Federation of Gynaecology and Obstetrics (FIGO) Preconception Checklist [2], along with Awareness and utilization of PCC, with a focus on vaccination and lifestyle domains, sources of health information, and perceived barriers to PCC. Anthropometric measurements were taken using standard procedures, and blood pressure was recorded using an automated sphygmomanometer. Blood samples for the field were collected for testing of HbA1C and TSH levels. The HbA1C tests were carried out by HIND labs using TOSHO G11 equipment (Tosho, Tokyo, Japan) with the same reagent kits along with BIORAD diabetic QC kit using TOSHO calibrator. Same labs using COBAS Pure equipment with Roche kits and Randox level 1,2,3 QC kits. The Cobos calibrator kit was used for calibration. 

Data were entered in Microsoft Excel and analyzed using IBM SPSS Statistics for Windows, Version 29 (Released 2024; IBM Corp., Armonk, New York, United States). Continuous variables were summarized as mean (±SD) or median (IQR), depending on distribution. Categorical variables were expressed as proportions (%). Bivariate associations between PCC awareness and independent variables were tested using χ² tests. Variables with p < 0.20 in bivariate analysis were included in multivariable logistic regression to identify independent predictors of PCC awareness. Odds ratios (OR) with 95% confidence intervals (CI) were reported. A p-value < 0.05 was considered statistically significant.

## Results

A total of 220 married nulligravida women participated in the study. The majority were aged ≤25 years 130(59.1%) and were housewives 180(81.8%). Most women had been married for more than one year, 185 (84.1%). Educational attainment varied: 110(50.0%) up to/below high school, and 110(50.0%) studied above high school level. Nearly 153 (75.5%) of participants had an income of >=3944 INR. Nearly 94 (42.7%) women had a BMI of >25 and <18.5% and 126 (57.3%) had normal BMI (18.5-24.9). Only 49 women (22.3%) had ever heard of PCC. Awareness of vaccination-related PCC was very low, 14(6.4%). Lack of knowledge was the most frequently cited reason for not accessing PCC services (≈98%). Even among women aware of PCC, actual service uptake remained limited: only 19 (38.8%) reported receiving counselling, investigations, or supplements. Most participants reported sedentary lifestyles, with dietary or exercise modifications rarely mentioned. Stress levels varied, but more than half, 127 (57.7%), reported moderate stress (level 2 on a graded scale) (Tables 1, 2).

**Table 1:** Distribution of the study participants by socio-demographic and morbidity characteristics (n=220)

Socio-demographic Details	N(%)
Age(years) of the study participants	
<=25	130(59.1)
>25	90(40.9)
Occupation	
Housewife/not working	180(81.8)
Working	40(18.2)
Years of marriage	
<=1	35(15.9)
>1	185(84.1)
Education level	
Up to/below high school	110(50)
above high school	110(50)
SES (n=217) [*3 participants not provided information about socioeconomic status]	
<=3943 INR	64(24.5)
>=3944 INR	153(75.5)
BMI (kg/m^2)	
Normal (18.5-24.9)	126(57.3)
<18.5 />25	94(42.7)
Know case of a disease	
No	195(88.6)
Hydrosalpinx	1(0.5)
PCOD	5(2.3)
Anemia	3(1.4)
Hyperthyroidism	1(0.5)
Hypothyroidism	10(4.5)
Others	5(2.3)
History of Surgery in past	
No	218(99.1)
Yes	2(0.9)
Stress level	
1	18(8.2)
2	127(57.7)
3	48(21.8)
4	16(7.3)
5	3(1.4)
6	1(0.5)
7	4(1.8)
8	2(0.9)
9	1(0.5)

**Table 2:** Distribution of study participants according to socio-demographic, health, and behavioral characteristics in relation to awareness about preconception care (n=220) PCC: Preconception care; SES: socioeconomic status; IFA: iron and folic acid

Variable	Category	Have You Heard the Word PCC?	
		Yes N(%)	No N(%)
Age (years)	<=25	32 (65.3)	98(57.3)
	>25	17 (34.7)	73(42.7)
Occupation	Housewife/not working	34 (69.4)	146(85.4)
	Working	15(30.6)	25(14.6)
Education	up to/below high school	20 (40.8)	90(52.6)
	above high school	29(59.2)	81(47.4)
Years of marriage	<=1	11(22.5)	24(14.0)
	>1	38(77.5)	147(86.0)
Total family numbers	<=2	6(12.2)	9(5.3)
	>2	43(87.8)	152(88.9)
SES	<=3943 INR	9(14.0)	55(86.0)
	>=3944 INR	40(25.8)	115(74.2)
known case of disease	No	39(79.6)	156(91.2)
	Yes	10(20.4)	15(8.8)
Source of information regarding PCC	No	4(8.2)	153(89.4)
	Newspaper/health care professional	45(91.9)	18(10.5)
Benefits of PCC	No	23(46.9)	166(97.1)
	Better pregnancy outcomes, IFA supplementation	26(53.1)	5(2.9)
Components of PCC	Not aware	36(73.5)	167(97.7)
	IFA supplementation, diet and exercise	13(26.5)	4(2.3)
Participants have health insurance	No	12(24.5)	49(28.7)
	Yes	37(75.5)	122(71.4)
Services received by participants	None	30(61.2)	153(89.5)
	Counselling, Evaluation, Investigations, IFA supplementation	19(38.8)	18 (10.5)
Reasons for not accessing PCC	Lack of knowledge	48(98.0)	167(97.7)
	Felt no need	1(2.0)	4(2.3)
Health behavior influenced by biomarker screening results	No change in health behavior	43(87.8)	163(95.3)
	Dietary modifications, exercise	6(12.2)	8(4.7)
Barriers for accessing PCC	Lack of knowledge/awareness	48(98.0)	171(100.0)
	No barriers	1(2.0)	0(0.0)
BMI (kg/m^2)	Normal (18.5-24.9)	26(53.1)	100(58.5)
	<18.5 />25	23(47.0)	71(41.5)

Elevated HbA1c (>=6.5%) was observed in 14 (8.2%) of women unaware of PCC, compared with 3(6.1%) among those aware but with no statistical significance (p ≈ 0.06) and χ²=3.536. The majority of women had TSH ≤ 4.0mIU/L. Abnormal TSH values were seen in 12 (7%) of unaware and 3 (6.1) of aware participants, with no significant difference between groups (p > 0.80) and χ²=0.048 (Table 3).

**Table 3:** Association between biomarker levels (HbA1c and TSH) and preconception care awareness (n=220) TSH: Thyroid-stimulating hormone; PCC: preconception care

Bio-marker	Category	Awareness of PCC		Total	Chi-square	Degrees of Freedom (df)	Odd’s Ratio (95% CI)	p-value
		Yes N (%)	No N (%)					
HbA1C (%)	>=6.5	3(6.1)	14(8.2)	17 (7.7)	3.536	1	2.178(0.954-4.972)	0.06
	<6.5	46 (93.9)	157(91.8)	203 (92.3)				
TSH (mIU/L)	>4	3 (6.1)	12(7.0)	15 (6.8)	0.048	1	1.157(0.313-4.276)	0.827
	<=4	46 (93.9)	159(93)	205 (93.2)				

Table 4 shows that 32 (65.3%) participants aged ≤25 and 17 (34.7%) participants (34.7%) aged >25 had knowledge of PCC, with no significant difference (p=0.316). Housewives/not working 34 (69.4%) had higher odds of PCC knowledge (OR 2.576, p=0.011), but this lost significance after adjustment (p=0.233). Those with education above high school 29 (59.2%) and longer marriage >1 year 38 (77.6%) showed no significant effect. Most had >2 family members 43 (87.8%). PCC knowledge was strongly linked to the source of information (45 (91.8%), OR 95.6, p<0.001); benefits awareness (26 (53.1%), OR 37.5, p<0.001); PCC components knowledge (13 (26.5%), OR 15.1, p<0.001; and immunization awareness 14 (28.6%), OR 5.9, p<0.001). Lack of knowledge was the major barrier (48; 97.96%) with a significant impact (p=0.049).

**Table 4:** Association of socio-demographic and health-related factors with knowledge about preconception care among study participants (n=220) PCC: Preconception care

Variable	Category	Knowledge about PCC Yes (%)	Chi square	Degrees of Freedom	Odds Ratio	p-value	Adjusted Odds Ratio	p-value
Age(years)	<=25	32 (65.31)	1.007	1	0.713 (0.368 - 1.382)	0.316	..	..
	>25	17 (34.69)						
Occupation	Housewife/not working	34 (69.39)	6.548	1	2.576 (1.288 - 5.405)	0.011	0.349 (0.062-1.968)	0.233
	Working	15 (30.61)						
Education	Up to/below high school	20 (40.82)	2.127	1	1.611 (0.846 - 3.067)	0.145	1.007 (0.289 - 3.510)	0.991
	Above high school	29 (59.18)						
Years of marriage	<=1	11 (22.45)	2.015	1	0.564 (0.254 - 1.252)	0.156	5.192 (0.692-38.931)	0.109
	>1	38 (77.55)						
Total no. of family numbers	<=2	6 (12.24)	0.049	1	0.896 (0.337 - 2.383)	0.826	..	..
	>2	43 (87.76)						
Socioeconomic status	<=3943	9 (18.36)	3.597	1	2.126 (0.963 - 4.69)	0.058	..	..
	>=3944	40 (81.6)						
Known comorbidity	No	39 (79.59)	5.12	1	2.667 (1.113 - 6.389)	0.024	0.494 (0.105-2.965)	0.494
	Yes	10 (20.41)						
Source of information regarding PCC	No	4 (8.16)	123.216	1	95.625 (30.79 - 296.985)	<0.001	0.007 (0.001-0.035)	<0.001
	Newspaper/health care professional	45 (91.84)						
Benefits of PCC	No	23 (46.94)	79.088	1	37.53 (13.11 - 107.438)	<0.001	0.048 (0.007 - 0.334)	0.002
	Better pregnancy outcomes, IFA supplementation	26 (53.06)						
Components of PCC	Not aware	36 (72.47)	31.26	1	15.076 (4.646 - 48.922)	<0.001	0.767 (0.068 - 8.658)	0.83
	IFA supplementation, diet and exercise	13 (26.53)						
Participants health insurance	No	12 (24.49)	0.33	1	1.238 (0.596 - 2.571)	0.586	..	..
	yes	37 (75.51)						
Services received by participants who have access to PCC	None	30 (61.22)	21.726	1	5.383 (2.532 - 11.44)	<0.001	1.925 (0.296 - 12.525)	0.493
	Counseling, Evaluation, Investigations, IFA supplementation	19 (38.78)						
Reasons for not accessing PCC	Lack of knowledge	48 (97.96)	0.015	1	0.87 (0.0095- 7.966)	0.061	0.064 (0.004-0.987)	0.049
	Felt no need	1 (2.04)						
Health behavior influenced by biomarker screening results	No change in health behavior	43 (87.76)	3.659	1	2.843 (0.936-8.632)	0.056	0.154 (0.010 - 2.420)	0.183
	Dietary modifications, exercise	6 (12.24)						
Barriers to accessing PCC	Lack of knowledge/awareness	48 (97.96)	3.506	1	0.219 (0.171-0.281)	0.902	..	..
	No barriers	1 (2.04)						
Immunization	Aware of vaccination-related care	14 (28.57)	52.178	1	5.886 (4.352 - 7.96)	<0.001	..	..
	Not aware	35 (71.43)						

The odds ratio (OR) for awareness of PCC in the higher HbA1c category was 2.18 (95 % CI: 0.95-4.97; p = 0.06). This indicates that participants with HbA1c ≥ 6.5 % had about 2.2 times higher odds of being aware of PCC compared to those with HbA1c < 6.5 %; however, this association did not reach statistical significance. The chi-square value was χ² = 0.048, and the OR was 1.16 (95 % CI: 0.31-4.28; p = 0.827) for TSH and PCC awareness. No statistically significant association was found between elevated TSH (>4mIU/L) and awareness of PCC.

## Discussion

Summary of the key findings

Our community-based cross-sectional study done in 220 nulliparous women discusses about unmet need for PCC among married nulliparous women in Guntur, Andhra Pradesh. Sociodemographic patterns showed that housewives/non-working women were more aware of PCC than working women (OR = 2.576, p = 0.011). Although educational status was not statistically significant (p = 0.087), a positive trend was evident. Access to healthcare professionals and media was the strongest predictor of PCC awareness (OR = 95.625, p < 0.001). The biomarker analysis showed no statistically significant association. Women with elevated HbA1c (≥6.5%) had higher odds of PCC awareness (OR = 2.178), although this trend did not reach significance (p = 0.060). This suggests that women already engaged in healthcare-likely due to metabolic risk-may have slightly greater exposure to health information, but this opportunity is not being consistently used to deliver PCC counselling. TSH levels showed no meaningful association with awareness (OR = 1.157, p = 0.827). Our study found that three-fourths of participants had never heard of PCC, indicating a critical knowledge gap. The unawareness of vaccination-related PCC represented the most significant. The study also revealed important associations between PCC knowledge and healthier behaviors, suggesting that education and awareness interventions could yield substantial improvements in preconception health outcomes. Out of those aware, more than half had never received any PCC services, underscoring gaps in service availability and delivery. Nearly all participants cited “lack of knowledge” as the main barrier to accessing PCC.

Comparison with existing literature

Doke et al. (2021) [13] reported less PCC awareness among rural Indian women, while Kumari and Kumar (2025) [14] found low awareness among both tribal and non-tribal women in a qualitative assessment. However, this study provides the first quantitative estimate of this knowledge gap, specifically among nulliparous women in Andhra Pradesh. Lack of knowledge regarding PCC reported in our study population is similar to those reported in other low- and middle-income countries, namely Ayle et al. (2017) [15] found that 72.5% of women in Northwest Ethiopia lacked adequate PCC knowledge, while Alie et al. (2022) [12] reported that only 28.6% of women in Ethiopia utilized PCC services. The systematic review by Poels et al. (2016) [16] identified poor awareness as a global barrier to PCC utilization, but the current study suggests that this barrier may be particularly pronounced in rural and semi-rural Indian communities. Finding that access to healthcare professionals and media sources was the strongest predictor of PCC knowledge (OR = 95.625, p <0.001) highlights the critical importance of information access in determining health knowledge. This finding is consistent with research by Chutke et al. (2022) [17], who identified healthcare worker engagement as crucial for PCC implementation. The concentration of unawareness among housewives suggests that traditional gender roles may limit women's access to health information and decision-making autonomy regarding preconception health, and this finding is consistent with qualitative research by Doke et al. (2021) [13]. The study found PCC-aware participants had not received any PCC services, highlighting significant gaps in healthcare service delivery, and is consistent with research by Stephenson et al. (2018) [4]. The morbidity profile of women in our study is similar to the findings of Chowdary et al. [18], which included anemia, obesity, hypothyroidism, etc. In the Indian context, this finding reflects the absence of structured PCC programs within the public health system, despite policy recognition of their importance. In the study, 93.6% of participants were unaware that vaccination-related PCC represents the most significant service gap. This finding is particularly concerning given the WHO's 2024 article [19] that emphasizes vaccination before and during pregnancy as a key component of PCC. The magnitude of this gap suggests that vaccination programs in India may not be effectively reaching women in the preconception period. The integration of vaccination counselling into PCC services represents a critical intervention point that is currently underutilized.

Interpretation

The higher rates of PCC unawareness in the current study may reflect regional differences in healthcare communication, cultural factors specific to South India, or the particular vulnerabilities of nulliparous women who have not yet engaged with maternal healthcare services. The substantial knowledge gap identified has several important implications. First, it suggests that the reproductive health continuum in India lacks adequate preconception components. Second, it indicates that current maternal and child health programs may be missing opportunities for primary prevention among women before their first pregnancy. The WHO emphasis on PCC as a strategy to reduce maternal and childhood mortality appears not to have been effectively translated into awareness and action at the community level in this setting. This disconnect between international recommendations and local implementation reflects broader challenges in health system strengthening and community engagement that have been identified across South Asian countries. This comparison highlights the substantial global inequities in PCC awareness and the need for context-specific interventions in low-resource settings.

The concentration of unawareness among housewives suggests that community-based interventions targeting women in domestic settings may be more effective than facility-based approaches alone. This finding supports calls for training healthcare providers in PCC counselling and developing systematic approaches to delivering PCC information through existing healthcare channels. This includes developing standardized protocols, training materials, and supportive supervision systems to ensure consistent and high-quality PCC services.

Strengths and limitations

Strengths

This study provides one of the first quantitative estimates of PCC awareness among nulliparous women in Andhra Pradesh. The community-based design enhances the representativeness of the findings. Identification of strong predictors (occupation, access to health professionals/media) provides actionable evidence for targeted interventions.

Limitations

The cross-sectional design prevents conclusions about causality between determinants and PCC awareness. Self-reported data may be subject to recall or social desirability bias, especially regarding health behaviors.

The study focused on select biochemical markers and did not capture the full spectrum of health indicators related to PCC.

The limitation also suggests the need for longitudinal, multi-site studies with broader biomarker assessments to better understand PCC awareness and utilization.

Implications for public health practice and policy

The study highlights an urgent need to integrate PCC more effectively into India’s reproductive health programs, like the introduction of community-level PCC awareness campaigns targeting homemakers. Integration of PCC services into existing maternal and child health programs. Training the grassroots-level workers, like ASHA workers, by developing structured PCC protocols and training modules.

## Conclusions

The study revealed that understanding and use of PCC among women of reproductive age in Andhra Pradesh remain inadequate. Only about one in four women demonstrated adequate awareness, and service utilization was considerably lower. Women who were employed, belonged to higher socioeconomic groups, or had previous exposure to health information showed greater awareness, indicating that information access and social engagement play important roles in shaping preconception health knowledge. The borderline association between elevated HbA1C and PCC awareness indicates that metabolic risk factors may influence women’s perception period and reproductive health needs.
